# Research on Coordination of Fresh Produce Supply Chain in Big Market Sales Environment

**DOI:** 10.1155/2014/873980

**Published:** 2014-02-09

**Authors:** Juning Su, Jiebing Wu, Chenguang Liu

**Affiliations:** ^1^School of Economics and Management, Xi'an University of Technology, Xi'an 710048, China; ^2^College of Public Administration, Zhejiang University, Hangzhou 310058, China

## Abstract

In this paper, we propose two decision models for decentralized and centralized fresh produce supply chains with stochastic supply and demand and controllable transportation time. The optimal order quantity and the optimal transportation time in these two supply chain systems are derived. To improve profits in a decentralized supply chain, based on analyzing the risk taken by each participant in the supply chain, we design a set of contracts which can coordinate this type of fresh produce supply chain with stochastic supply and stochastic demand, and controllable transportation time as well. We also obtain a value range of contract parameters that can increase profits of all participants in the decentralized supply chain. The expected profits of the decentralized setting and the centralized setting are compared with respect to given numerical examples. Furthermore, the sensitivity analyses of the deterioration rate factor and the freshness factor are performed. The results of numerical examples show that the transportation time is shorter, the order quantity is smaller, the total profit of whole supply chain is less, and the possibility of cooperation between supplier and retailer is higher for the fresh produce which is more perishable and its quality decays more quickly.

## 1. Introduction

Fresh produce, such as fresh fruits, fresh vegetables, fresh flowers, and live seafood, characteristically has a random lifetime in the postharvest period. The decay/deterioration risk creates huge uncertainties for the effective supply and demand of fresh produce. As a result, both suppliers and retailers involved in the supply chain could suffer substantial losses. For this reason, coordination of the supply chain plays an important role in the supply chain management of fresh produce [1]. This is especially true in the “big market sales” environment. A market condition in which the supplier and retailer are far apart is referred to as a “big market sales” environment in this paper.

Trade in fresh produce has been among the most dynamic areas of the international agriculture trade, stimulated by rising incomes and growing consumer interest in product variety, freshness, convenience, and year-round availability. Advances in production, postharvest handling, processing, and logistical technologies, along with increased levels of international investment, have played a facilitating role. China is currently the world's largest producer of fruits and vegetables [2]. China exported 4.795 million tons of fruit and 9.73 million tons of vegetables to foreign markets in 2011. The big market sales mode is common in the international fresh produce trade because the supplier and retailer are far from one another. In such an environment, quantity deterioration, quality decay, and the transportation costs of fresh produce in delivering the product from its origin to the target sales market become considerations that cannot be ignored in the supply chain.

This paper attempts to analysis coordination of the fresh produce supply chain in a big market sales environment. When the supply chain involves long-distance transportation, shortening logistics times may decrease the possible product decay/deterioration, but it may require expensive transportation. Therefore, the supply chain for fresh produce needs to determine the best tradeoff between the associated costs and benefits to optimize the total profit. Decay/deterioration decreases not only the effective supply but also the freshness level that might impact market demand (i.e., market demand depends on the freshness level). The research questions are threefold: how should a decision-maker optimize order quantity and logistics time? How are the optimal order quantity and logistics time determined? Could one develop an effective mechanism under which both parties can be better off? In so doing, we first give a quantitative description about the perishability properties of fresh produce and the relationship between decay/deterioration losses and time. Then, we analyze quantitatively transportation costs in a big market sales environment and the relationship between transportation costs and time. Based on this, we investigate decision-making models under both decentralized and centralized supply chain settings and analyze solutions to the models. Further, based on a comparative analysis of the risks and benefits faced by the parties, we put forward a design idea for a coordinated scheme. Finally, a combined incentive contract is developed using supply chain coordination theory.

The remainder of this paper is organized as follows. In Section 2, we review the related literature. In Section 3, we describe the problem and the notation. In Section 4, we construct decision models of decentralized and centralized supply chains for fresh produce, and optimal order quantity and logistics time in the two supply chain systems are derived.  Section 5 is dedicated to the development of a coordination contract in the fresh produce supply chain.  Section 6 illustrates the sensitivity analysis of parameters in the models.  Section 7 concludes and outlines areas for future research.

## 2. Literature Review

This research is most related to the literature in two different areas: models for perishable product ordering and inventory control and coordination models for a perishable product supply chain.

Numerous models for managing the ordering and inventory of perishable products have been developed. Ghare and Schrader [3] were the first researchers to consider decaying inventory. They develop an economic order quantity model for products in which the number of usable units is subject to exponential decay when demand is constant. Covert and Philip [4] extend this model to use a Weibull distribution to describe item deterioration. Nahmias [5] computes the optimal inventory policy for a product with a multiperiod shelf life when ordering, holding, run-out, and out-dating costs are considered. Tadikamalla [6] examines the model for items with gamma distribution deterioration. Elasayed and Teresi [7] developed the optimal order level for deteriorating items. Nahmias [8] gives a thorough review of the early literature on perishable inventory and classifies the perishable product into fixed lifetime and random lifetime types. Fixed lifetime perishable items are those that can be stored for a specified fixed time, and after that time, they must be discarded. However, random lifetime perishable items are those that will be discarded after an uncertain expiration time. Subsequently, Kalpakam and Arivarignan [9], Rau et al. [10], and Ghosh et al. [11] consider ordering and/or inventory models where the items deteriorate continuously at a constant perishing rate. Wee [12] studies perishable product ordering strategy under quantity discounts and buyback, with the assumption that the deterioration rate obeys a two-parameter Weibull distribution. Halim et al. [13] discuss economic order quantities in cases where the product deterioration rate is fuzzy.

In the most early research on perishable inventory, perishability is defined as the number of units of product that are outdated (perish); thus, decay is not in terms of quality or value. However, in recent studies, some papers propose models that address deterioration in terms of reduction of product quantity and degradation of quality and value over time. Weiss [14] examines a situation where the value of an item decreases nonlinearly the longer it is held in stock. Fujiwara and Perera [15] develop EOQ models for inventory management under the assumption that product value diminishes over time according to an exponential distribution. More recently, Ferguson et al. [16] apply Weiss' model to optimal order quantities for perishable goods in small-to-medium size grocery stores with delivery surcharges. Blackburn and Scudder [17], using the product's marginal value of time (MVT), the rate at which the product loses value over time, develop a model to minimize lost value. In the most recent study, Sainathan [18] examines both inventory and pricing control of perishable products by taking into account that the quality of the “new” product is higher than that of the “old” product. Using this information, we extend the conventional EOQ model to the ordering model for fresh produce in a big market sales environment, which is characterized by simultaneously considering quantity loss and quality decay, including logistics costs and random demand and supply; the optimal order quantity and logistics time are derived.

To date, ordering and inventory control models for perishable products have been extended to the supply chain. Yang and Wee [19] developed an integrated deteriorating inventory model for both buyers and vendors; the integrated approach results in an impressive costreduction compared with independent decisions by the buyer. Sarker et al. [20] develop supply chain models to determine an optimal ordering policy for deteriorating items under inflation, permissible delay of payment, and allowable shortage. Rau et al. [10] develop a multiechelon inventory model for a deteriorating item and to derive an optimal joint total cost from an integrated supply chain perspective among the supplier, the producer, and the buyer. Subsequently, a coordination model of the supply chain for perishable product is investigated. Poole et al. [21] survey a fruit vendor and retailer in Spain and obtain some important factors that affect fresh produce supply chain cooperation with an empirical analysis. Weng [22] developed a framework to address the problem of coordinating decisions of the manufacturer and the distributor operating to meet price-sensitive random demand for a product with a short product life cycle. Ferguson and Ketzenberg [23] examine the value of information sharing, considering a supplier sharing age-dependent information with retailers for perishable products. Ketzenberg and Ferguson [24] studied the value of sharing the retailer's information on aging and demand with the supplier. Further, Xu [25] investigates the optimal ordering and pricing decisions of suppliers and distributors considering the uncertainty of long-distance transportation. Nahmias [26] recently reviews the literature on the perishable goods supply chain with models that consider different aspects (e.g., random versus deterministic lifetime, stochastic versus deterministic demand).

Although many ordering and inventory models of perishable products have been developed, there are few reports that study the supply and demand coordination problem of fresh produce from the perspective of the supply chain. Among many reports, the focus is on perishable products with fixed lifetimes (i.e., the marketing life or shelf life of the products although it is relatively short but is a certain period.), such as high technology products, fashion apparel, medicines, and food products, but few discuss perishable products with random lifetime (i.e., the duration in which the products are alive or useful is relatively short but is a uncertain period), such as fresh produce. However, fresh produce, for example, fresh fruits and fresh vegetables, can be found anywhere as part of our daily life. As a result, there is a continued need to understand fresh produce and to investigate the impact of perishability and the random life of products on supply chain decisions. Thus, this study particularly aims to develop ordering models for such a system to explore optimal coordination contract in the supply chain, considering deterioration and long-distance transportation in the context of big market sales. Our study differs from existing studies mainly in the following three aspects. First, we derive random effective supply rate expressions for fresh produce based on our insight that there is a variety of other complex factors affecting the life-span of fresh produce beyond simply time. Second, we develop a joint decision model that includes ordering and delivering decision-making. Third, we design a combined contract form that can achieve supply chain coordination for fresh produce. To our knowledge, this is the first reported study that examines both ordering and delivery control of fresh produce, taking into account random demand and supply and variable transportation time.

## 3. Problem Description and Model Assumptions

### 3.1. Problem Description

This paper studies a two-level supply chain system for fresh produce, consisting of a supplier (such as a production base or a professional cooperative in the original) and a retailer (such as a dealer or exporter in the distant market). Such a supply chain is a specific case of serial supply chains [27]. The supplier cleans, sorts, packages, processes, and supplies fresh produce in accordance with an order from the retailer. The supplier has a very large supply capacity and cannot run out of stock. The retailer is responsible for shipping the fresh produce to distant market to sell. The transportation market is well developed from the place of origin to the sale market and there is a wide variety of logistics solutions to choose from for the retailer.

**Figure 1:**
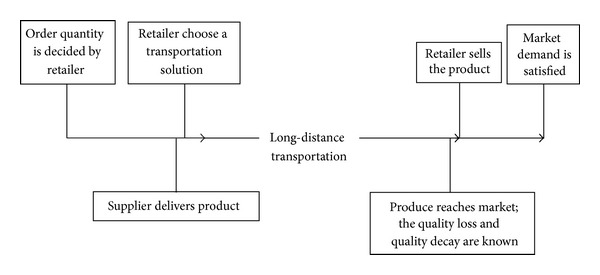
Timeline of decisions in the fresh produce supply chain.

Because fresh produce is a class of highly perishable products, the longer the transportation time during the long-distance transportation process is, the more the deteriorating produces are. This results in a more quantity loss of fresh produce and a smaller effective supply of fresh produce reaching the target market. Meanwhile, the longer the transportation time is, the more the serious quality decay of fresh produce is, and the smaller the freshness of fresh produce is. This has a greater impact on market demand. Thus, the retailer is motivated to shorten the logistics time. The retailer can control transportation time by selecting different transportation solutions. However, shortening transportation time tends to increase transportation costs because an extra urgent transportation expense is paid. And, the more the transportation time is compressed, the faster the urgent delivery costs rise. Thus, the decision-maker needs to weigh the two aspects of transportation: time and economy. The decision-making sequence for the supply chain of fresh produce is shown in Figure 1.

As a kind of perishable product, we assume that the salvage value of unsold fresh produce is zero, and the shortage cost of fresh produce is not taken into account. Furthermore, we assume that both parties of supply chain share information, are risk-neutral, and pursuing expected profit maximization.

### 3.2. Notation and Assumptions

Consider the following:

*c*_*s*_: unit production cost of the supplier;
*w*: wholesale price of the supplier;
*q*: order quantity of the retailer, a decision variable;
*t*: transportation time from origin to sales point, a decision variable for the retailer (retailer controls transportation time by choosing different transportation methods). We assume that *t* ∈ [*t* ^*l*^, *t* ^*u*^], where *t* ^*u*^ is the normal transportation time and *t* ^*l*^ is the minimum possible transportation time;
*p*: the retail price of the retailer;
*θ*(*t*): the deterioration rate of fresh produce; it increases with transportation time. *θ*(*t*)∈[0,1], *θ*′(*t*) > 0; label 1 − *θ*(*t*) as *m*(*t*) indicates the effective supply factor of fresh produce;
*M*(*t*, *ε*_1_): effective supply rate when product reaches the target market; it is decreased by the transportation time and influenced by the random factor *ε*_1_, *M*(*t*, *ε*_1_)∈[0,1];
*λ*(*t*): freshness level of the fresh produce. As transportation time lengthens, freshness declines. 0 ≤ *λ*(*t*) ≤ 1, *λ*′(*t*) < 0. The freshness level equals 1 when the fresh produce is in its freshest state. We assume that the freshness of the produces that retailer loads is 1. From the beginning of transportation, the freshness level of fresh produces decreases gradually;
*c*: normal transportation fee. With given starting and ending points, the normal transportation cost is related to the quantities of fresh produce transported, decided as a unit transportation fee *c* multiplied by order quantity *q*;
*v*(*t*): urgent transportation cost; this refers to extra cost caused by the retailer choosing urgent transportation so as to shorten the transportation time, to reduce deterioration loss during transportation. Urgent transportation costs are related to the degree of compression of the transportation time;
*c*(*t*): total transportation cost;
*D*(*p*, *λ*(*t*), *ε*_2_): market demand of the product, influenced by product price *p*, freshness level *λ*(*t*), and random factor *ε*_2_;
Π_*s*_: the expected profit of the supplier;
Π_*r*_: the expected profit of the retailer;
Π: total expected profit of the supply chain.

In the following models, subscript *f* indicates a decentralized uncoordinated system, subscript *j* indicates a centralized supply chain system, subscript *x* indicates a decentralized coordinated system, and superscript ∗ indicates the optimal value.

We propose several basic assumptions before modeling.

**Assumption 1**

*M*(*t*, *ε*_1_) = *m*(*t*)*ε*_1_, *ε*_1_ is a random factor of continuous distribution; its mean is 1, and the probability density function and distribution function are *g*(*x*) and *G*(*x*), respectively. This function indicates the effects of other random factors apart from transportation time (e.g., temperature or humidity and human factors, such as handling, loading and unloading, theft, and loss.) on the fresh produce effective supply rate.

**Assumption 2**

The market demand for fresh produce is either affected by the sales price or the freshness level of produce. With the same price, the higher the freshness level of produce is, the bigger the market demand is. With the same freshness level of produce, the lower the price is, the bigger the market demand is. Take a multiplicative form of market demand: *D*(*p*, *λ*(*t*)) = *ap* ^−*k*^ *λ*(*t*)*ε*_2_, where *ε*_2_ is a random factor of continuous distribution, its mean is 1, and the probability density function and distribution function are *f*(*x*) and *F*(*x*), respectively. The function indicates the effects of other random factors except for price and freshness level on market demand. The *a* measures the size of the market and is a constant. The *k* is price elasticity, *k* > 1, so that the lower the price, the greater the demand.

**Assumption 3**

Consider *c*(*t*) = *cq* + *v*(*t*), *v*(*t*) = (1/2)*v*(*t* ^*u*^ − *t*)^2^, where *v* is time cost coefficient. Transportation cost includes two parts: normal transportation costs and urgent transportation costs. The latter one increases quickly with the degree of time compression.

## 4. Decisions in a Decentralized and Centralized Supply Chain

### 4.1. Model and Solutions for a Decentralized Supply Chain

In a decentralized supply chain, the retailer makes decisions independently about the order quantity and transportation time by the way of maximizing its profit. The supplier makes the choice between accepting or rejecting the retailer's decisions. Then, the expected profit functions of retailer, supplier, and supply chain system in a decentralized supply chain are, respectively, as follows:

(1) $$\begin{array}{c} \Pi_{rf}\left(q,t\right)=pE\left\{\min\left\{M\left(t\right)q,D\left[p,\lambda \left(t\right)\right]\right\}\right\}-qw-c\left(t\right), \end{array}$$

(2) $$\begin{array}{c} \Pi_{sf}=qw-qc_{s}, \end{array}$$

(3) $$\begin{array}{c} \Pi_{f}\left(q,t\right)=pE\left\{\min\left\{M\left(t\right)q,D\left[p,\lambda \left(t\right)\right]\right\}\right\}-qc_{c}-c\left(t\right). \end{array}$$

In the case where both effective supply and market demand follow a random distribution, the optimal decision for the retailer should aim to maximize the expected profit statistically. There are two random variables, *ε*_1_ and *ε*_2_, in the retailer's profit function (1). When the fresh produces reach the distant markets, the effect of random variable *ε*_1_ on effective supply of fresh produces can be observed (Figure 1). Then, (1) can be solved in two steps: first, we fix *ε*_1_ and only consider the effect of random variable *ε*_2_ on the retailer's profit. Then, we consider the effect of random variable *ε*_1_ on the retailer's profit. Thus, in the decentralized setting, the retailer's model is max⁡Π_*rf*_(*q*, *t*) = *E*_*ε*_1__[Π_*rf*_(*q*, *t* | *ε*_1_)] when *ε*_1_ equals *ξ*_1_,

(4) Πrf(q,t ∣ ξ1) =pE{min⁡⁡{m(t)ξ1q,D[p,λ(t)]}}−qw−c(t).

According to Petruzzi and Dada [28], define an inventory factor: *z* = *m*(*t*)*ξ*_1_ *q*/[*ap* ^−*k*^ *λ*(*t*)], substituting it into (4); then, (4) can be rewritten as

(5) Πrf(z ∣ q,t,ξ1) =(zaλ(t)m(t)ξ1q)1/kEε2{min⁡⁡{m(t)ξ1q,m(t)ξ1qzε2}}  −qw−cq−v(t).

According to Ferguson and Ketzenberg [23], optimal inventory factor, *z*_0_, is uniquely decided by equation ∫_0_^*z*^(*k* − 1)*xf*(*x*)*dx* = *z*[1 − *F*(*z*)]. It can be observed that *z*_0_ has no relationship with *q* or *t*; therefore, by substituting *z*_0_ and simplifying (5), we get

(6) Πrf(q,t ∣ ξ1)=(z0aλ(t))1/k[m(t)ξ1q](k−1)/k ×Eε2{min⁡⁡{1,ε2z0}}−qw−cq−v(t)=(z0aλ(t))1/k[m(t)ξ1q](k−1)/k ×(1−∫0z0(1−xz0)f(x)dx) −qw−cq−v(t)=(z0aλ(t))1/k[m(t)ξ1q](k−1)/kk(1−F(z0))k−1 −qw−cq−v(t).

Based on (6) and using random variable *ε*_1_, we can derive the expected profit function of the retailer, as follows:

(7) Πrf(q,t)=Eε1[Πrf(q,t ∣ ε1)]=kk−1A0q(k−1)/kλ(t)1/km(t)(k−1)/k −qw−cq−v(t),

where *A*_0_ = (*az*_0_)^1/*k*^[1 − *F*(*z*_0_)]*E*_*ε*_1__{*ε*_1_^(*k*−1)/*k*^}.

**Proposition 4**

In decentralized supply chain, when the transportation time of fresh produce from the origin to the sales point is given by *t*, the retailer's optimal order quantity is *q*_rf_*(*t*) = *λ*(*t*)*m*(*t*)^*k*−1^(*A*_0_/(*w*+*c*))^*k*^.

**Proof**

In (7), we fix *t* and obtain a first-order differential and a second-order differential with regard to *q*:

(8) ∂Πrf∂q=Aoλ(t)1/km(t)(k−1)/kq−1/k−w−c,∂2Πrf∂q2=−1kA0λ(t)1/km(t)(k−1)/kq−(k+1)/k.

It can be observed that ∂^2^Π_*rf*_/∂*q* ^2^ < 0, so Π_*rf*_ is strictly a concave function with regard to *q*. For a given *t*, there exists a *q*_rf_*(*t*) that makes Π_*rf*_ maximal at this point. Let ∂Π_*rf*_/∂*q* = 0; then, we can obtain

(9) $$\begin{array}{c} q_{rf}^{*}\left(t\right)=\lambda \left(t\right)m{\left(t\right)}^{k-1}{\left(\frac{A_{0}}{\left(w+c\right)}\right)}^{k}. \end{array}$$

Substituting *q*_rf_*(*t*) into (7), the retailer's expected profit function with regard to transportation time *t* can be obtained:

(10) $$\begin{array}{c} \Pi_{rf}\left(t\right)=\frac{A_{0}^{k}\lambda \left(t\right)m{\left(t\right)}^{k-1}}{\left(k-1\right){\left(w+c\right)}^{k-1}}-v\left(t\right). \end{array}$$

On the one hand, compressing the transportation time can reduce product deterioration and increase the effective supply on reaching the market; more fresh product and bigger market demand can increase the retailer's income simultaneously. On the other hand, compressing the transportation time will increase the urgent transportation costs. Thus, there must exist an optimal transportation time that maximizes the retailer's profit.

The first-order differential of Π_*rf*_(*t*) with regard to *t* is

(11) dΠrf(t)dt=A0k(k−1)λ(t)m(t)k−2m′(t)+A0kλ′(t)m(t)k−1(k−1)(w+c)k−1−v′(t).

Because we cannot judge whether *d*Π_*rf*_(*t*)/*dt* is greater than zero and cannot know the points which make the value of *d*Π_*rf*_(*t*)/*dt* equal to zero also, the monotonicity and the stationary points of the function Π_*rf*_(*t*) cannot be known, and so it is impossible to directly get optimal transport time *t*_rf_*. However, according to different situations, we can finally get *t*_rf_* by classification discussion. The *t*_rf_* is given by Proposition 5 as follows.

**Proposition 5**

In the decentralized supply chain, the retailer's optimal transportation time can be obtained according to the following approach:

 
*when d*Π_*rf*_(*t*)/*dt* < 0, *t*_rf_* = *t* ^*l*^ *;*
 

*when d*Π_*rf*_(*t*)/*dt* > 0, *t*_rf_* = *t* ^*u*^ *;*
 

*otherwise, t*_rf_* = argmax {Π_*rf*_(*t* ^*l*^), Π_*rf*_(*t*_1_), Π_*rf*_(*t*_2_),…Π_*rf*_(*t*_*n*_), Π_*rf*_(*t* ^*u*^)}*, in which *{*t*_1_, *t*_2_, *t*_3_,…*t*_*n*_}* is the solution set of the equation d*Π_*rf*_(*t*)/*dt* = 0.

Substituting *t*_rf_* into (9), we can obtain the retailer's optimal order quantity:

(12) $$\begin{array}{c} q_{rf}^{*}=\lambda \left(t_{rf}^{*}\right)m{\left(t_{rf}^{*}\right)}^{k-1}{\left(\frac{A_{0}}{w+c}\right)}^{k}. \end{array}$$

Substituting *t*_rf_* into (10), we can obtain the retailer's optimal expected profit under a decentralized system:

(13) $$\begin{array}{c} \Pi_{rf}^{*}=\frac{A_{0}^{k}\lambda \left(t_{rf}^{*}\right)m{\left(t_{rf}^{*}\right)}^{k-1}}{\left(k-1\right){\left(w+c\right)}^{k-1}}-v\left(t_{rf}^{*}\right). \end{array}$$

Substituting *q*_rf_* into (2), we can obtain the supplier's optimal profit:

(14) $$\begin{array}{c} \Pi_{sf}^{*}=\lambda \left(t_{rf}^{*}\right)m{\left(t_{rf}^{*}\right)}^{k-1}{\left[\frac{A_{0}}{w+c}\right]}^{k}\left(w-c_{s}\right). \end{array}$$

The expected profit of the whole supply chain is

(15) Πf∗=A0kλ(trf∗)m(trf∗)k−1(k−1)(w+c)k−1−v(trf∗)+λ(trf∗)m(trf∗)k−1[A0w+c]k(w−cs).

### 4.2. The Model and Solutions of Centralized Supply Chain

In centralized supply chain, the supplier and the retailer are an interest unit and they cooperate closely, sharing information with each other and pursuing total profit maximization as their objective. The expected profit of supply chain is

(16) $$\begin{array}{c} \Pi_{j}\left(q,t\right)=pE\left\{\min\left\{M\left(t\right)q,D\left[p,\lambda \left(t\right)\right]\right\}\right\}-qc_{s}-c\left(t\right). \end{array}$$

Similar to procedure in Section 4.1, the model of centralized supply chain can be written as

(17) max⁡Πj(q,t)=Eε1{Πj(q,t ∣ ε1)}=kk−1A0q(k−1)/kλ(t)1/km(t)(k−1)/k −qcs−cq−v(t).

Similar to methods in Section 4.1, the optimal order quantity of centralized supply chain can be obtained as Proposition 6.

**Proposition 6**

In the centralized supply chain, when the transportation time of fresh produce from the origin to the sales point is given as *t*, the optimal order quantity of supply chain is

(18) $$\begin{array}{c} q_{j}^{*}\left(t\right)=\lambda \left(t\right)m{\left(t\right)}^{k-1}{\left(\frac{A_{0}}{c_{s}+c}\right)}^{k}. \end{array}$$

Substituting *q*_j_*(*t*) into (17), the expected profit function of the supply chain with regard to transportation time *t* can be obtained:

(19) $$\begin{array}{c} \Pi_{j}\left(t\right)=\frac{A_{0}^{k}\lambda \left(t\right)m{\left(t\right)}^{k-1}}{\left(k-1\right){\left(c_{s}+c\right)}^{k-1}}-v\left(t\right). \end{array}$$

Similarly, optimal transportation time *t*_j_* can be obtained as Proposition 7.

**Proposition 7**

In the centralized supply chain, the optimal transportation time of supply chain can be obtained according to the following approach:

1. *if d*Π_*j*_(*t*)/*dt* < 0*, then t*_j_* = *t* ^*l*^ *;*
2. *if d*Π_*j*_(*t*)/*dt* > 0*, then t*_j_* = *t* ^*u*^ *;*
3. *else, one solves the equation d*Π_*j*_(*t*)/*dt* = 0* and then labels the solution set as T*_*j*_ = {*t*_*j*1_, *t*_*j*2_, *t*_*j*3_,…*t*_*jn*_}.

Then, *t*_j_* = argmax {Π_*j*_(*t* ^*l*^), Π_*j*_(*t*_*j*1_), Π_*j*_(*t*_*j*2_),…, Π_*j*_(*t*_*jn*_), Π_*j*_(*t* ^*u*^)}.

Substituting *t*_j_* into (18), we can obtain the optimal order quantity under a centralized supply chain:

(20) $$\begin{array}{c} q_{j}^{*}=\lambda \left(t_{j}^{*}\right)m{\left(t_{j}^{*}\right)}^{k-1}{\left[\frac{A_{0}}{c_{s}+c}\right]}^{k}. \end{array}$$

Substituting *t*_j_* into (19), we can obtain the optimal expected profit under a centralized supply chain:

(21) $$\begin{array}{c} \Pi_{j}^{*}=\frac{A_{0}^{k}\lambda \left(t_{j}^{*}\right)m{\left(t_{j}^{*}\right)}^{k-1}}{\left(k-1\right){\left(c_{s}+c\right)}^{k-1}}-v\left(t_{j}^{*}\right). \end{array}$$

Because the models are too complex, the explicit formulations of the optimal transport time and those of the optimal order quantity in the decentralized setting and centralized setting cannot be obtained. But we can know that the optimal decisions of the retailer in the decentralized setting are different from those of the whole supply chain in the centralized setting by intuitively observing. The optimal decisions of these two situations will be compared via numerical example in Section 6.1.

Now we compare the optimal order quantity in the decentralized setting with that in the centralized setting based on assuming that the optimal transport times in these two situations all are *t* ^*u*^. In Proposition 4, *q*_rf_* = *λ*(*t* ^*u*^)*m*(*t* ^*u*^)^*k*−1^(*A*_0_/(*w* + *c*))^*k*^ when *t* = *t* ^*u*^. In Proposition 6, *q*_j_* = *λ*(*t* ^*u*^)*m*(*t* ^*u*^)^*k*−1^(*A*_0_/(*c*_*s*_ + *c*))^*k*^ when *t* = *t* ^*u*^. Because *w* > *c*_*s*_, *q*_rf_* < *q*_j_* when *t* = *t* ^*u*^. That is, the optimal order quantity of the retailer in decentralized setting is less than that of the whole supply chain in the centralized setting.

We compare the total profit of supply chain in the decentralized setting and that in the centralized setting. Because the (*q*_j_*, *t*_j_*) is the point which maximizes the function Π_*j*_(*q*, *t*), there exists Π_*j*_(*q*, *t*) ≤ Π_*j*_(*q*_j_*, *t*_j_*) for any point (*q*, *t*). It also holds for Π_*j*_(*q*_rf_*, *t*_rf_*) ≤ Π_*j*_(*q*_j_*, *t*_j_*). Comparing (3) with (16), it is obvious that the total profit function forms of supply chain in the decentralized and centralized setting are exactly consistent, so it holds that Π_*f*_(*q*_rf_*, *t*_rf_*) = Π_*j*_(*q*_rf_*, *t*_rf_*). Because Π_*j*_(*q*_rf_*, *t*_rf_*) ≤ Π_*j*_(*q*_j_*, *t*_j_*) and Π_*f*_(*q*_rf_*, *t*_rf_*) = Π_*j*_(*q*_rf_*, *t*_rf_*), Π_*f*_(*q*_rf_*, *t*_rf_*) ≤ Π_*j*_(*q*_j_*, *t*_j_*). That is, the total profit of supply chain in decentralized setting is less than that in centralized setting.

## 5. Coordination of a Decentralized Supply Chain

In reality, the decentralized supply chain is more common. Thus, it is necessary to implement a coordination mechanism for a decentralized supply chain, so as to the decisions for order quantity and transportation time made from the point of view of the retailer are consistent with the optimal decisions for the supply chain to realize supply chain optimization. In designing the coordination mechanism, the decisions of centralized setting are often used as a benchmark for the decentralized system to reach coordination.

### 5.1. Design of the Coordination Contract

Supply chain contracts are a common supply chain coordination mechanism. The design principles of the supply chain coordination mechanism are risk sharing and revenue sharing. Then, we have to analyze the risks borne by the supplier and the retailer in a decentralized system. It is obvious that, in an uncoordinated decentralized systems, the risks of supply and market demand uncertainty caused by product deterioration are both passed on to the retailer, so it is necessary for the supplier to share some of the risks in designing a coordination contract to motivate the retailer to order more products. According to this thinking, this paper proposes the following combined contracts.

(*1) A Wholesale Price Discount Contract.* Because of the decaying of fresh produce, the effective supply of product decreases. We can consider this as an increase in procurement cost for the retailer (or as an increase in wholesale price for the supplier) in disguise. A wholesale price discount contract would be adopted to make the supplier share some risk caused by the deterioration of the produce, and this would stimulate the retailer to order more produces.

The design idea of the wholesale price discount contract is that the supplier adopts cost-plus pricing method to determine the list wholesale price which means that the list wholesale price equals *c*_*s*_ to add a *φ* proportion of marginal profit of per unit product in supply chain. When the sales price of fresh produce is *p*, the supply chain marginal profit obtained from per unit of produce is *p* − *c* − *c*_*s*_. Then the list wholesale price of supplier can be written as *w*_0_ = *c*_*s*_ + *φ*(*p* − *c* − *c*_*s*_). When the produces reach the target market and part of produces decay, the wholesale price should be cut down based on the list wholesale price so as to make the supplier share some part of losses from produce deterioration. If the real effective supply rate is *m*(*t*)*ξ*_1_, the deterioration loss of unit produce is *p* − *m*(*t*)*ξ*_1_ *p*. Given the deterioration loss share ratio of the supplier is *φ*; then the wholesale price of supplier will reduce *φ*(*p* − *m*(*t*)*ξ*_1_ *p*) based on the list wholesale price *w*_0_. So the specific form of the wholesale price function offered by the supplier is

(22) $$\begin{array}{c} w\left(t\right)=w_{0}-\varphi \left[p-m\left(t\right)\xi_{1}p\right]. \end{array}$$

Substituting the expression of *w*_0_ into (22), we can obtain the wholesale price discount contract as follows:

(23) $$\begin{array}{c} w\left(t\right)=c_{s}+\varphi \left[m\left(t\right)\xi_{1}p-c-c_{s}\right]. \end{array}$$

The wholesale price discount contract connects the interests of supplier with the interests of retailer by establishing relationships between wholesale price and retail price. So the supplier shares the risks with the retailer together under a wholesale price discount contract.

(*2) Unsaleable Produce Subsidy Contract.* Uncertain market demand brings an unmarketable product risk. This risk is borne by the retailer when there is not coordination contract. We design a contract in which the supplier shares some risk of unsaleable produce by providing a certain percentage of subsidies for losses due to unsold produce. Because fresh produce is perishable, we assume that the salvage value for surplus produce is zero at the end of the sales period. For every unsold produce, the retailer will lose *p*, and the supplier renders *φp*. Then the amount of subsidy *s* is

(24) $$\begin{array}{c} s=\varphi p. \end{array}$$

(*3) Cost-Compensating Contract.* Urgent transportation can shorten transportation time, so it can reduce the deterioration of the fresh produce and can keep the fresh produce fresh. All these effects benefit product sales, but the retailer needs to pay the extra urgent transportation costs. Therefore, we propose a cost-compensating contract that makes the supplier provide a portion of *φ* towards the retailer's urgent transportation costs. The symbol *z* indicates the amount of compensation given by the supplier. The form of the cost-compensation contract is

(25) $$\begin{array}{c} z=\varphi v\left(t\right). \end{array}$$

### 5.2. Analysis of Decision-Making and Coordination under Combined Contracts

**Proposition 8**

In coordination with combined contracts, *w*(*t*) = *c*_*s*_ + *φ*[*m*(*t*)*ξ*_1_ *p* − *c* − *c*_*s*_], *s* = *φp*, and *z* = *φv*(*t*), the retailer's optimal order quantity and optimal transportation time are consistent with optimal decisions of the centralized supply chain.

**Proof**

With the combined contracts which consist of the three contracts above, the expected profit function of the retailer can be transformed into

(26) Πrx(q,t)=Eε1[Πrx(q,t ∣ ε1)]=Eε1{pEε2{min⁡⁡{m(t)ξ1q,D[p,λ(t)]}}−qw(t)−c(t)+sEε2×{{m(t)ξ1q−D[p,λ(t)]}+}+z}.

The above function can be expanded as follows:

(27) Πrx(q,t)=Eε1{p{m(t)ξ1q−Eε2{m(t)ξ1q−D[p,λ(t)]}+} −qw(t)−c(t)+sEε2 ×{{m(t)ξ1q−D[p,λ(t)]}+}+z}=Eε1{[m(t)ξ1p−w(t)−c]q−(p−s)Eε2×{{m(t)ξ1q−D[p,λ(t)]}+}−v(t)+φv(t)}=Eε1{(1−φ){[m(t)ξ1p−cs−c]q−pEε2×{{m(t)ξ1q−D[p,λ(t)]}+}}−(1−φ)v(t)}=(1−φ)Eε1 ×{pEε2{min⁡⁡{m(t)ξ1q,D[p,λ(t)]}}−qcs−c(t)}=(1−φ)Eε1{Πj2(q,t ∣ ε1)}=(1−φ)Πj(q,t).

Obviously, under the combined contracts, the optimal decisions of the retailer are suboptimization of the decisions of the entire supply chain.  Proposition 8 is proven.

It can be demonstrated that the retailer's optimal profit is (1 − *φ*)Π_j_*, and the supplier's optimal profit is *φ*Π_j_*. This shows that *φ* not only represents the proportion that the supplier shares of the risks of the supply chain under a combined contract but also represents the proportion that the supplier obtaines of the total profits of the entire supply chain. This illustrates that the combined contracts designed for a fresh produce supply chain embody a profit distribution principle in risk market; that is, the greater the risk, the greater the returns.

**Proposition 9**

The combined contracts can achieve perfect coordination of the supply chain for fresh produce when *φ* belongs [*φ*_min⁡_, *φ*_max⁡_], where

(28) φmin⁡=A0k[λ(trf∗)m(trf∗)(cs+c)]k−1(w−cs)(k−1) ×((w+c)k[A0kλ(tj∗)m(tj∗)k−1−(k−1)(cs+c)k−1v(tj∗)])−1,φmax⁡=1−(((cs+c)k−1[A0kλ(trf∗)m(trf∗)k−1−(k−1)(w+c)k−1v(trf∗)])×((w+c)k−1[A0kλ(tj∗)m(tj∗)k−1−(k−1)(cs+c)k−1v(tj∗)])−1).

**Proof**

Proposition 8 illustrates that, under combined contracts, decentralized supply chain performance reaches the performance level of the centralized system. To realize Pareto improvement, with the members of the supply chain both accepting this contract, the following two conditions must be satisfied Π_rx_* = (1 − *φ*)Π_j_* ≥ Π_rf_*, and Π_sx_* = *φ*Π_j_* ≥ Π_sf_*.

Through mathematical derivation, the following results can be obtained:

(29) φmin⁡ =A0k[λ(trf∗)m(trf∗)(cs+c)]k−1(w−cs)(k−1)  ×((w+c)k[A0kλ(tj∗)m(tj∗)k−1−(k−1)(cs+c)k−1v(tj∗)])−1,φmax⁡ =1−((cs+c)k−1[A0kλ(trf∗)m(trf∗)k−1−(k−1)(w+c)k−1v(trf∗)]×((w+c)k−1[A0kλ(tj∗)m(tj∗)k−1−(k−1)(cs+c)k−1v(tj∗)])−1).

Because *φ*_max⁡_ − *φ*_min⁡_ = 1 − Π_rf_*/Π_j_* − Π_sf_*/Π_j_* = [Π_j_* − (Π_rf_* + Π_sf_*)]/Π_j_* > 0, the interval [*φ*_min⁡_, *φ*_max⁡_] exists and Proposition 9 is proven.

When *φ* = *φ*_min⁡_, all increased profits in supply chain coordination are occupied by the retailer, while the profit increment for the supplier is 0. The retailer is absolutely dominant in the supply chain. In contrast, when *φ* = *φ*_max⁡_, all increased profits in supply chain coordination flow to the supplier, while the profit increment for the retailer is 0, and the supplier is in the dominant position of supply chain. Thus, when the value of *φ* is given in [*φ*_min⁡_, *φ*_max⁡_], the purpose can achieve that the coordination profit of supply chain is discretionarily allotted between the parties of the supply chain. The practical value of *φ* depends on the relative bargaining power of the parties.

## 6. Numerical Examples

The model expressions in Sections 4 and 5 are complex, and we cannot obtain the explicit solutions. In order to illustrate the proposed models, we give numerical examples as follows. Consider a fresh produce supply chain with the following characteristics: *c*_*s*_ = 4, *w* = 6, *p* = 12. The deterioration characteristics accord with a traditional three-parameter Weibull function, *θ*(*t*) = *αβ*(*t* − *γ*)^*β*−1^, where *α* = 0.1, *β* = 1.1, and *γ* = 0.5. Freshness function is *λ*(*t*) = *λ*_0_^*t*^, *λ*_0_ = 0.999. The parameters of the transportation method from the origin to market are as follows: *c* = 1, *t* ^*u*^ = 10, *t* ^*l*^ = 5, and *v* = 500. Other values are as follows: *k* = 2, *a* = 500000, *ε*_1_ ∈ *U*[0,2], *ε*_2_ ∈ *U*[0,2]. According to Ferguson and Ketzenberg [23], the optimal inventory factor satisfies the following equation:

(30) $$\begin{array}{c} z_{0}=\frac{4}{k+1},\;\;F\left(z_{0}\right)=\frac{2}{k+1}. \end{array}$$

### 6.1. Solution of the Models

Substituting these parameters into the models and computing with Matlab, we can then obtain the optimal decisions and each party's profits in decentralized and centralized systems (Table 1).

It can be concluded from Table 1 that the order quantity in the centralized system is larger than that in the decentralized system, the transport time in the centralized system is shorter than that in the decentralized system, and the overall profit of the whole supply chain in the centralized system is higher than that in the decentralized system. However the implementation conditions of the centralized system are harsh; it is common to see the decentralized system in reality. After introducing the combined contracts proposed in this paper, the coordination conditions can be calculated as *φ* ∈ [0.2041,0.2857], in which contract can be accepted by both the supplier and the retailer, and it can make the overall profit of the decentralized supply chain reach the level of that of the centralized supply chain. When contract parameter *φ* gets value in this range, the changes in the profits and its increments of the retailer and the supplier after coordination with the parameter *φ* are shown in Table 2.

It can be concluded from Table 2 that the profits of the supplier and the retailer after coordination increase than those before coordination when *φ* is within the scope of valid value of it. With an increasing of the value of *φ*, the profit increments of the retailer are declining while the profit increments of the supplier are increasing, and the increased profits of supply chain after coordination transfer from the retailer to the supplier gradually. These verify that the combined contract can coordinate fresh produce supply chain effectively, and the combined contract can flexibly allocate the increased profits of supply chain after coordination between the supplier and the retailer in an arbitrary ratio, when *φ* is within the scope of valid value of it.

### 6.2. Sensitivity Analysis of the Models

To further analyze the adaptability of the models and to provide more management implications for the fresh produce supply chain in practice, in this section, we aim to analyze the impact of several important model parameters on decision-making results.

#### 6.2.1. Influence of Deterioration for Fresh Produce on Decision-Making

**Figure 2:**
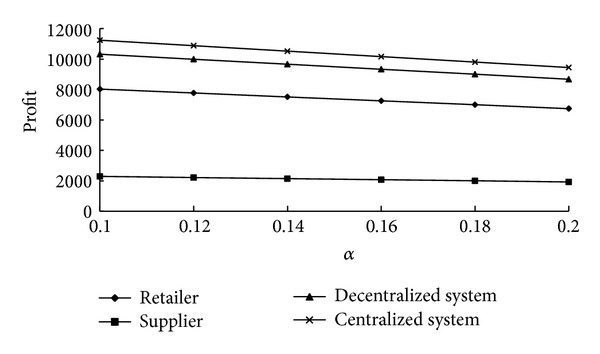
Profits of supply chain parties with deterioration factor *α*.

The perishability nature is one of the most important characteristics of fresh produce. In the context of big market sales, quantity loss of fresh produce, caused by long distance transportation, occurs due to decay. How does the deterioration characteristic of the fresh produce affect the decisions in the supply chain? In this section, we perform a sensitivity analysis of parameter *α* which comes from the deterioration rate function. The larger the value of *α* is, the more perishable the produce is, and the more the quantity loss of the fresh produce during transportation is. When the value of *α* changes in [0.1, 0.2], the optimal decisions of decentralized and centralized systems and the value range of supply chain coordination parameter *φ* are shown in Table 3.  Figure 2 shows that the profits of all parties and supply chain change with *α* either in decentralized system or in centralized system.

We can make the following conclusions by analyzing Table 3 and Figure 2.

**Observation 1**

Whether in a decentralized or centralized system, the more perishable the produce is, the shorter the transportation time is, and the smaller the order quantity is. This observation can be explained because the more perishable the produce is, the greater the potential loss is, and the decision-maker is therefore more cautious.

**Observation 2**

As *α* becomes larger, the profits of every party and total profit of supply chain tend to decrease in a decentralized system; also, the profit of the entire supply chain decreases gradually in a centralized system. This observation illustrates that the more perishable the produce becomes, the weaker the profitability of supply chain is.

**Observation 3**

For supply chain coordination, as *α* becomes bigger, the lower and upper limits of the contract parameter *φ* increase at the same time; however, the upper limit increases faster than the lower limit, and the value range of *φ* becomes bigger and bigger. This observation illustrates that the more perishable the produce is, the higher the possibility of cooperation between supplier and retailer is.

#### 6.2.2. Influence of Quality Decay for Fresh Produce on Decision-Making

**Figure 3:**
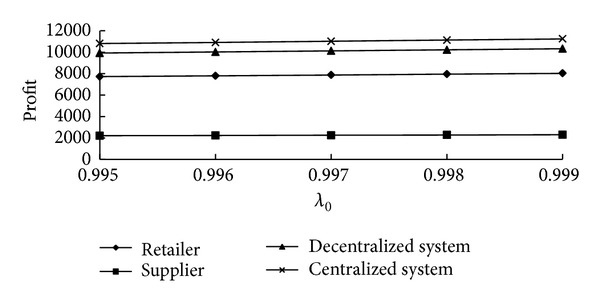
Profits of supply chain parties with freshness factor *λ*_0_.

Apart from its perishable nature, freshness is another important characteristic of fresh produce. So, how does freshness affect decisions in the supply chain? Previously, we used a freshness level function to describe the freshness of fresh produce. The parameter *λ*_0_ in the function indicates fresh-keeping performance. The larger *λ*_0_ is, the easier the produce is to keep fresh. When the value of *λ*_0_ changes in [0.995, 0.999], we analyze decision results of different supply chain systems and coordination conditions in Table 4.  Figure 3 shows that the profits of all parties and supply chain change with freshness factor *λ*_0_ either in decentralized system or in centralized system.

We can make the following conclusions by analyzing Table 4 and Figure 3.

**Observation 4**

As freshness factor *λ*_0_ becomes bigger, the optimal order quantity and transportation time both become bigger in both decentralized and centralized supply chains. This observation illustrates that the easier the produce retains fresh, the more of the produce the retailer tends to order, and therefore the retailer selects a cheaper transportation method.

**Observation 5**

As freshness factor *λ*_0_ becomes larger, the profits of every party and total profit of supply chain increase at the same time in the decentralized system, and the profit of the centralized system increases also. This observation illustrates that a produce that decays more slowly is beneficial to all parties in the supply chain.

**Observation 6**

As freshness factor *λ*_0_ becomes larger, the lower and upper limits of contracts parameter *φ* decrease simultaneously; however, the upper limit decreases faster, so the value range of *φ* becomes smaller. This observation illustrates that the faster the quality of produce decays, the higher the possibility of cooperation between supplier and retailer is.

## 7. Conclusions

In the context of the rapid development of modern agriculture and logistics, the “big market sales” model of fresh produce sales has prevailed. This paper constructs a deterioration rate function and freshness function for fresh produce that depend on the transport time in long-distance transportation. It is assumed that effective supply is an indeterminate variable influenced by deterioration rate and random factors and that market demand is a random variable influenced by price and freshness level, random factor as well. Based on these assumptions, decision models of decentralized and centralized supply chains are built and we present a solution algorithm for the models. By analyzing numerical examples, we find that order quantity in a centralized system is higher than that in a decentralized system, while transportation time in a centralized system is shorter than that in a decentralized system and the total profit of the supply chain in a centralized system is higher than that in a decentralized system. We design a combined contract to coordinate the decentralized supply chain which consists of a wholesale price discount contract, an unsaleable produce subsidy contract, and a cost-compensating contract. A mathematical derivation demonstrates that the combined contracts can effectively coordinate a two-level supply chain of fresh produce, where supply and demand both conform to a time-varying random distribution, and can discretionarily allot the coordination profit of the supply chain between the supplier and retailer. Then, we provide the conditions with which the coordination of supply chain is achieved. Using a sensitivity analysis of two important parameters (deterioration rate *α* and freshness factor *λ*_0_), we draw the conclusion that the more perishable the produce is, the faster the quality of produce decays, and the higher the possibility of cooperation between supplier and retailer is. These conclusions provide a better understanding of fresh produce supply chain management practices.

Our study makes some contributions to the understanding of integrated optimization of more than that in a decentralized procurement and logistics in the fresh produce supply chain. Another contribution of our work is the design of a combined contract which ensures that both parties are better off by coordinating in a situation where both the effective supply and the market demand of the produce are random; freshness, deterioration rate, and transportation costs are sensitive to time, and market demand is sensitive to freshness level.

Although this study provides several managerial implications for fresh produce supply chains, the paper only studies quantity loss and quality decay of fresh produce caused by long-distance transportation and assumes that freshness only affects market demand. Freshness also affects the market price of fresh produce, and the value of fresh produce would therefore be reduced over long-distance transportation. Additionally, the implementation of the combined contracts proposed in this paper requires that there is mutual cooperation and information sharing between the supplier and retailer. Such conditions are difficult in practice. Thus, we suggest further research to study supply chain coordination of fresh produce under conditions of asymmetric information.

## Figures and Tables

**Table 1:** Optimal decisions and profits in decentralized and centralized systems.

	q*	t*	Π_r_*	Π_s_*	Π*
Decentralized decision	1147.2	9.96	8030	2294	10324
Centralized decision	2248.6	9.94	—	—	11242
Δ*q*/Δ*t*/ΔΠ	1101.4	0.02	—	—	918

**Table 2:** The profits and their increments of the retailer and supplier after coordination.

*φ*	Π_rx_*	ΔΠ_*r*_ = Π_rx_* − Π_rf_*	Π_sx_*	ΔΠ_*s*_ = Π_sx_* − Π_sf_*
0.2041	8948	918	2294	0
0.2245	8718	688	2524	230
0.2449	8489	459	2753	459
0.2653	8259	229	2983	689
0.2857	8030	0	3212	918

**Table 3:** Optimal decisions in each supply chain system with deterioration factor α.

α	Decentralized system		Centralized system		Coordination system		
	*q*_rf_*	*t*_rf_*	*q*_j_*	*t*_j_*	*φ*_min⁡_	*φ*_max⁡_	Δ*φ*
0.10	1147.2	9.957	2248.6	9.940	0.2041	0.2857	0.0816
0.12	1110.6	9.952	2176.9	9.933	0.2042	0.2858	0.0816
0.14	1074.0	9.947	2105.1	9.926	0.2042	0.2859	0.0817
0.16	1037.3	9.942	2033.4	9.919	0.2044	0.2861	0.0817
0.18	1000.7	9.937	1961.7	9.912	0.2045	0.2864	0.0819
0.20	964.1	9.932	1889.9	9.905	0.2048	0.2868	0.0820

**Table 4:** Optimal decisions in each supply chain system with freshness factor λ_0_.

λ_0_	Decentralized system		Centralized system		Coordination system		
	*q*_rf_*	*t*_rf_*	*q*_j_*	*t*_j_*	*φ*_min⁡_	*φ*_max⁡_	Δ*φ*
0.995	1102.7	9.90	2162.0	9.85	0.2041	0.2858	0.0817
0.996	1113.7	9.91	2183.2	9.88	0.2040	0.2856	0.0816
0.997	1124.7	9.93	2204.7	9.90	0.2040	0.2856	0.0816
0.998	1135.9	9.94	2226.6	9.92	0.2039	0.2854	0.0815
0.999	1147.2	9.96	2248.6	9.94	0.2037	0.2850	0.0813
